# Additional Provision for the Insane

**Published:** 1909-01

**Authors:** F. S. White

**Affiliations:** Formerly Superintendent of the State Lunatic Asylum, Austin, Texas, and Assistant Superintended North Texas Hospital for the Insane, Terrell, Texas


For Texas Medical Journal.

### Additional Provision for the Insane.

BY F. S. WHITE, TERRELL, TEXAS.
Formerly Superintendent of the State Lunatic Asylum, Austin, Texas, and Assistant Superintenden North Texas Hospital for the Insane, Terrell, Texas.

"Canst thou not minister to a mind diseased,
Pluck out the memory of a rooted sorrow,
Raze out the written trouble of the brain
And with some sweet oblivious antidote
Cleanse the stuffed bosom of that perilous stuff
Which weighs upon the heart?"

One of the most important problems that confronts a civilized nation is the proper care of its helpless, defective and useless population. This class represents those who from either inherent or acquired weakness have fallen by the wayside. They have failed in the battle of life and are now only worthless burdens on the more favored citizens. As the great current of civilization flows on at an ever-increasing rate, many fall out and line the way as driftwood that is forced to the shore of a swollen stream as it rushes to the sea. Humanity demands that these defectives be provided for, and in such a manner as to make their lives as pleasant as possible. The higher the state of civilization that a people attains the better they should care for those who have been unable to maintain their mental equilibrium and continue to be self-supporting. Many of these have been useful and happy citizens who contributed their part to the development of the country and to the support of the government. They have added somewhat to the material wealth of the community, and now in their hour of dire distress and helplessness they have a right to expect their country to come to their relief. Many of them are yet able under intelligent direction and supervision to aid very much in their own support, and, by so doing, render their lives more endurable, if not happy. A large majority of the insane in this country come from the farming and laboring classes. This is true because these interests largely predominate in our population. On account of this fact this enormous amount of energy and force should be utilized by intelligent supervision, in the direction of least resistance. The insane can be made very largely self-sustaining by judicious and up-to-date management. The plans for doing this have been worked out to a very high degree of perfection by older countries and States. Some most egregious and expensive errors have been made in the attempt to provide for the insane of Texas. In this line of work as in every other, advantage should be taken of the results attained by countries that have already passed through the expensive stage of experimenting in this direction.

When additional provision for the insane is made it should not be of a temporary character, but should be of that kind which experience and time have shown to be best, and the best is not always the cheapest at first.

The prime object of such provision is to cure the insane and enable them to return to their homes where they can again assume the pleasures and obligations of citizenship and be producers of wealth rather than purely consumers. As every insane person restored to mental health is a burden lifted from the competent and more favored citizens, every effort should be made to restore these unfortunates to their right minds and thus save the State from the expense of their support. These people who through no fault of their own have become an unwilling burden upon the State have the right to demand that they be given the best scientific treatment and the best possible chance to get well.

In the first place, the buildings provided for this class should be constructed on plans that make them the most conducive to recovery, and then they should be placed in charge of men whose study, experience and acquirements make them experts in this line of work.

There is something more to be done than simply to house, clothe and feed these patients. Their cases should be the object of close scientific study and treatment. The name "Asylums for the Insane" should be abolished and relegated to the superstitious past and "State Hospitals" substituted, and the fact emphasized that treatment is the main object for which patients are received. Jury trials for the insane—that relic of medieval ignorance and barbarism—should be stricken from our laws and more scientific procedures substituted. These people are not criminals or violators of the law that they should be arraigned before the courts of the country and "found guilty." They are only unfortunate victims of the saddest calamity that can befall a human being, and this calamity is brought about by disease, and they should be treated accordingly.

The past half a century has witnessed remarkable advancement and improvement in the care and treatment of the insane. Formerly they were supposed to be possessed of devils and evil spirits, but now they are known to be only mentally sick people, the cause of which is disease of the brain.

This period has witnessed their emergence from dark and gruesome cells and dungeons to more healthful and commodious buildings. This change has been brought about as a result of scientific study and research by experts in this line of work and not by time-serving politicians and officials. Science does not count the cost of such advancement in dollars and cents, but in the lives and happiness of human beings. As men of scientific experience have been responsible for the magnificent improvements made in behalf of this class of our defective and dependent population, such men should be consulted when matters affecting the insane are under consideration by lawmakers.

The welfare of these unfortunate people should be the prime object sought and not the upbuilding of any town or section of the State or the advancement of the political aspirations of any man or set of men. When it becomes necessary to provide additional provisions for the insane, the Legislature should lay aside all sectional feelings and broadly consider this important problem and do what is best for them regardless of any other influence.

Suppose an institution established according to the most modern and scientific plan does cost more than an old and obsolete plan, but if in it more mentally sick people can be cured and restored to useful citizenship and happy families and thereby relieve the State of a lifetime burden, is it not cheaper even in dollars and cents in the long run?

These people are entitled to the best possible chance to get well, and the grave responsibility of providing the means of restoration rests upon the State, and unless she provides the very best that is known to science she is derelict in her duty.

The time has again been reached when the insane have accumulated to such an extent in the jails and poorhouses that additional provisions must be made for them. Texas should now mature and adopt some permanent plan for the future care of the insane; a plan not to be carried out in one or two years, but to be executed as the demands arise.

Up to now it has been the policy of each administration to get through with the smallest possible expenditure of money, and, if possible, to reduce taxation, and the result has been that unsuitable and inefficient accommodation for the insane has been made with cheapness as the main object. The idea seems to have been to simply provide an asylum rather than an up-to-date hospital. This very fact is largely responsible for the increased accumulation of the insane.

Insanity is a very curable disease if given proper treatment and care in the early stages, but it is almost hopeless if neglected. I flatter myself in believing that the experience I have had in and the study I have given to the care and treatment of insanity fits me to some extent at least to make suggestions on this important matter.

The next Legislature will be called upon to consider this question, and I indulge the hope that it will immortalize itself by doing something in the right way in accordance with modern and scientific plans. My observation is that, as a rule, members of the Legislature are men who honestly desire to do what is best, and if they err it is on account of a lack of proper information.

To adopt and carry out the plans which I suggest will cost money; in fact, much more money than to go on adding to the present conglomeration of buildings already constructed without any definite and mature plan, but solely on account of cheapness. Texas is not a pauper State, and her magnanimous citizens will never complain at giving their unfortunate neighbors, relatives and friends the very best accommodations. If the Legislature wants to make cheap provision from a dollar standpoint for her insane, it can be done by adding to the institutions already built; but the ultimate results on the inmates will be far from satisfactory. The result of a hospital for the treatment of insanity should be measured by the recovery of the patients and not by the per capita cost for maintenance. Suppose it should cost a thousand dollars to restore a man in the early years of his manhood to mental health, is that not cheaper to the State than to care for him in a State institution for twenty or thirty years at a very low per capita expense without taking into consideration what he would produce for his country during that time?

Now, the plan which I have to suggest is similar to that in vogue in Scotland, which country is considered as having the best system in the world for treating the insane. Some of the older States of this country have adopted plans on the same order and have found them to be ultimately the cheapest and most successful. The present asylums of Texas can not be immediately changed, but they can gradually be systematized in accordance with modern ideas.

A tract of land consisting of several thousand acres should be acquired by the State. This should be good fertile land and the greater portion of it should be capable of a high state of cultivation. It should be in a healthy locality where crops are reasonably certain. There should be a bold flowing stream of water through it.

This land should also have on it stone in abundance and clay suitable for making brick. The scenery should be as varied as could be found, and there must be hills and valleys. The soil should be such that all kinds of grain, fruits, vegetables and flowers could be grown in great quantities. Some portion of this land should be subject to irrigation.

This entire tract should be carefully surveyed and mapped by an expert landscape gardner, assisted by experienced and scientific farmers, horticulturists and stockraisers. A definite and well-matured plan should be adopted to be carried out in future years as became necessary.

Upon this land should be erected three classes of buildings—one for the acute insane, one for the chronic insane and another class for the officers and employes. The buildings for the insane should be on what is known as the cottage plan; that is, small detached buildings, which for the chronic insane would accommodate from fifty to one hundred inmates. The buildings for the acute insane should be essentially hospitals with all necessary appliances and equipment to carry out this idea.

The buildings for the chronic and incurable cases should be constructed for comfort and convenience and be as homelike as possible. The asylum idea should be entirely eliminated. This entire magnificent plant to be under one competent management, whose every effort emphasized the fact that the sole object was to treat and cure insanity. No drones or incompetents should be permitted to encumber and retard the great work of the institution.

In the various industries of such a humanitarian and scientific colony all the patients who were physically able could be given employment, and in this way the cost of maintenance very materially reduced. Work and amusement are absolutely necessary to the successful treatment of insanity. At such an institution and amid such surroundings insane people would get well and, returning to their homes, become useful citizens.

It will be contended that to put such a plant in operation will necessitate a large outlay of money by the State. That is true, but it would not all be in one year, and the results would amply justify the expenditure. Why not adopt a plan that will take care of the insane for all time to come, and let the miserable makeshifts which have been built in the past be sufficient monuments to the folly and ignorance of short-sighted Legislatures?

The taxpayers of this great State are anxious for the insane to be well taken care of, and they will commend any expenditure of their money that is necessary for this purpose.

The humanitarian spirit of the citizenship of Texas demands that ample provision be made for the insane and that they be taken from loathsome jails and poorhouses.

It is a shame and disgrace that an unfortunate citizen of Texas should be forced to live in a jail or poorhouse covered with filth and associated with criminals.

Terrell, Texas, October, 1908.

